# Errors and Truth

**Published:** 1867-12

**Authors:** 


### ERRORS AND TRUTH

are alike exhumed from the grave of the past in mechanics, medicine and theology, and even in so-called "science" itself; the best remedy under the circumstances is for each man to be for himself "wary" of what is new, look into every thing proposed with a patient, close and critical eye, and never give up old things too readily; for in very many cases, our fathers were wiser in their generations than we sometimes give them credit for.

